# Older adults’ experiences of self-determination when needing homecare services—an interview study

**DOI:** 10.1186/s12877-023-04533-6

**Published:** 2023-12-08

**Authors:** Karin Bölenius, Kristina Lämås, David Edvardsson

**Affiliations:** 1 Department of Nursing, Umeå University, Umeå, 90187 Sweden; 2 School of Nursing and Midwifery, La Trobe University, Melbourne, Australia; 3 Sahlgrenska Academy, Institute of Health and Care Sciences, University of Gothenburg, Gothenburg, Sweden

**Keywords:** Decision-making, Homecare service, Interview, Involvement, Older adults, Personal autonomy, Self-determination

## Abstract

**Background:**

Self-determination has been shown to be an important factor in mental health and wellbeing, but from the homecare recipients’ point of view, autonomy and self-determination is not fully integrated into homecare services. The aim of this study was to explore older adults’ experiences of self-determination when needing homecare services.

**Methods:**

In 2018, a qualitative descriptive study was conducted and a convenience sample of 15 older adults from 3 homecare service facilities were invited to participate in individual interviews. Data were analysed using qualitative content analyse.

**Results:**

The theme *Transitioning from self-determination as independence towards self-determination as shared decision-making* emerged through the older adults’ narratives. This ‘transition’ is one in which older adult’s understanding of self-determination and self-esteem was transitioning towards the acceptance of shared decision-making. The person’s inner strength and willingness to make decisions was promoting to enact and preserve independence. Accepting one’s dependence on others and being in a positive atmosphere were described as promoting self-determination and shared decision-making, and vice versa. The above overarching theme permeated all subthemes, which included: *mobilising inner strength to enact independence; accepting increasing dependence on others;* and *being influenced by the atmosphere.*

**Conclusions:**

The study contributes increased understanding of older adults’ experiences of self-determination. The results can act as a guide when planning future person-centred care interventions in the context of homecare services and help improve homecare services’ ability to meet the needs of older adults. To summarise, older adults’ reflections on their own self-determination highlighted relationships with other people as important for shared decision-making, which could help preserve older adults’ autonomy and self-esteem.

## Background

In many parts of the Western world, an increasing number of older adults with weakened health are offered care at home [1–4]. Staying at home despite the need for care has been found to be preferable to moving to a nursing home [5]. One reason why older adults wish to remain at home has been found to be linked to the positive experience of autonomy and independence when living at home [4]. Autonomy is often understood as an individuals’ capacity to make their own decisions without the influence of others [6] and preserving autonomy has been shown to be an important factor in mental health and wellbeing [7]. A concept often used synonymously with autonomy is self-determination, which is defined as an individual’s control, legal and ethical rights, knowledge, and their ability to make decisions based on free choice [8]. Another concept related to self-determination is shared decision-making. Shared decision-making means that all relevant parties are involved in the entire decision-making process, they share information and preferences, and mutually agree upon a plan [9].

Living at home with assistance from homecare services (HCS) may be successful in terms of increased wellbeing. Older homecare recipients have described the following core values to be fundamental in homecare service encounters: being autonomous, self-determined, and establishing and maintaining relationships with care staff [10]. In addition, a cross-sectional study in Sweden [11] found that higher self-determination among HCS recipients was associated with higher health-related quality of life. Studies exploring self-determination that could serve as a guide when developing interventions that aim to increase self-determination among older adults are urgently needed. Additionally, and from the recipients’ point of view, autonomy and self-determination are not fully integrated into HCS [9]. In one interview study [12], nurses and older homecare recipients described homecare services as fragmented and organisationally driven. Care was given in a hasty way that was compared to assembly lines in a factory. To make it possible to continue to live at home, care designed around the individual was suggested, where the recipients of homecare receivers are listened to, supported in using their resources, and encouraged to take part in meaningful activities [12].

The Swedish Social Service Act [13] that regulates HCS states that people have a right to self-determination and integrity. In Sweden and worldwide, and in recent decades, the need for a person-centred approach in health care has been stressed [14, 15]. In person-centred care (PCC), the persons’ view of their life situation is in situated at the centre of care, which also includes the possibility of shared decision-making regarding the planning, prioritising, and performance of care [14]. In research about institutional care in a nursing and a multidisciplinary context, PCC has been reported to be associated with increased quality of life [16], and increased satisfaction with care among older adults [17]. PCC in HCS has been studied, to a limited extent. However, one study from Norway [18] among people with dementia living at home and receiving homecare explored the experience of PCC and decision-making. It was found to be unclear how exactly participants could participate in shared decision-making, and they seemed to have little influence on the care they received. At the same time, they seemed to adapt without question and appreciated being able to continue living at home.

Our research group has implemented a person-centred and health promoting intervention to increase older adults’ self-determination and involvement in care decisions when living at home and receiving homecare services [19]. The quantitative outcomes after the intervention [20] showed that despite increased self-reported negative emotions—i.e., depressed mood—participants in the intervention group continued to thrive while the control group showed no change in negative emotions, but they rated to thrive to a lesser extent. We therefore suggested that the PCC has a preservative effect on patients’ ability to thrive, despite deterioration. In addition, older adults with higher levels of self-reported self-determination also reported higher quality of life [11]. Knowledge about experiences of self-determination as a part of PCC in the context of HCS is limited, and further research is needed.

## Methods

The aim of this study was to explore older adults’ experiences of self-determination when needing homecare services.

This study is part of a HCS project that included an intervention based on the theoretical concepts of person-centredness, including self-determination and involvement in care decisions. A web-based educational program, supported by face-to-face seminars for diverse staff experiences, was implemented. Following the education, staff were directed to implement discussions with HCS recipients in the intervention group. Staff were also encouraged to maintain daily flexibility in PCC. The purpose was to maximise older adults’ health and to satisfy their psychosocial and physical needs [19, 20]. This article is a sub-study that will explore older adults’ experiences of receiving homecare after the intervention, with a focus on self-determination and involvement in care decisions.

### Design

A qualitative descriptive study was conducted based on individual interviews performed in 2018 investigating older adults’ experiences.

### Setting

This study was performed in a municipality in northern Sweden where the local HCS was mostly detail-driven and controlled via standardised schemas. The municipality in question had participated in a previous intervention study [20], and included three facilities. All included HCS facilities were publicly funded by taxes. In HCS, older adults’ care needs are assessed by a care manager who, based on the assessment, decides on the level of HCS needed [20]. In Sweden, staff working in HCS are, for example, responsible for cleaning, shopping, personal hygiene, assisting with physical activities, and household work [21].

### Participants

A convenience sample of older adults from the three HCS facilities that had been included in the intervention group [20] was invited to participate in the interview study. The older adults were living in either houses or apartments. The home care manager, along with the older adult’s contact person, assisted in identifying individuals who were willing to participate. All older adults (n = 15) initially approached for participation agreed and were included in this study. With 15 participants, data saturation was deemed achievable. In total, 10 participants were women, and 5 were men; all participants were aged 65 years or older. The oldest participant was 98 years old. Inclusion criteria for the older adults were having participated in the intervention group, being aged 65 years or older, living at home with assistance from HCS, and speaking and understanding Swedish. The sole exclusion criterion was suffering from any condition that impedes communication.

### Data collection and interviews

Potential participants received a formal written invitation with information reminding them about the study. Afterward, the authors (KB and KL) called the elderly individuals to inform them about the study verbally and inquire about permission to visit their homes and conduct interviews. Researchers offered the participants time to communicate about the written information they were given and schedule a date and time for the interview. The participants gave their written consent to participate before the interviews were conducted. Data were collected through narrative individual interviews.

Interviews were performed in the older adults’ homes and focused on their experiences of self-determination. Interviews were conducted by the two researchers (KB and KL) with experiences performing interviews and with knowledge of HCS. Open-ended questions were asked during the interviews. Examples of questions asked of the older adults were: Can you please tell me about your experiences of self-determination related to homecare staff visits? Can you please describe what you can and are allowed to decide related to the care you receive during homecare staff visits? Can you please describe what it means to you to be able to decide about HCS content? Can you please describe a situation in which you have felt self-determination? The interviews were recorded and transcribed verbatim. The interview time varied between 25 and 56 min.

### Analysis and interpretation

Data were analysed using qualitative content inspired by Lindgren et al. [22]. The transcribed text was first read through, after which the text was divided into meaning units. Text that did not meet the study aim was excluded. Meaning units were condensed and compared according to their content and then sorted and formulated into sub-themes and an overall theme. The structural analysis was validated by discussion in the research group, and discussions continued until consensus was reached. The analysis moved back and forth between meaning units and sub-themes to ensure a stringent and trustworthy interpretation of the data.

### Ethics

The study (Dnr 2016/04–31Ö) was approved by the Regional Ethics Review Board in Umeå, Sweden. The older adults involved in the study were informed about the project and told that their participation was voluntary. They were assured they were free to decline participation in the study at any time during the project. Participants were informed that confidentiality would be ensured in the presentation of the results and that no unauthorised person would have access to any of the study material. Authors experienced that the older adults were satisfied with the interview situation and happy to be involved in a research project. However, there is always a risk of the researcher–participant relationship becoming either too close or too distant [23].

## Results

Older adults’ narratives about their experiences of self-determination were revealed in the theme *transitioning from self-determination as independence to self-determination as shared decision-making.* In other words, older adult’s understanding of self-determination and self-esteem was transitioning to acceptance of shared decision-making. The person’s inner strength and willingness to make decisions was promoting to enact and preserve independence. Accepting one’s dependence on others and being in a great atmosphere were described as promoting self-determination and shared decision-making, and vice versa. The overarching theme permeated all subthemes, which included: *mobilising inner strength to enact independence; accepting increasing dependence on others;* and *being influenced by the atmosphere.* The theme and sub-themes are presented in Table 1, and the sub-themes presented in the results are validated with quotations from the interviews.

**Table 1:** Overview of overall theme and sub-themes

Sub-themes	Theme
Mobilising inner strength to enact independence as self-determination	Transitioning from self-determination as independence towards self-determination as shared decision-making
Accepting increasing dependence on others	
Experiencing self-determination as shared decision-making	

### Mobilising inner strengths to enact independence as self-determination

In summary, having inner strength enabled older adults to fight for their independence. This inner strength was described in terms of being content in oneself, thriving, and being strong. The capacity to mobilize this inner strength and make decisions that enact independence was facilitated by being resolute in one’s decisions, drawing from past experiences to make autonomous choices, and maintaining a willingness to decide and self-manage. With inner strength it became possible to express and make decisions that were sometimes described as uncomfortable.

Past experiences where individuals made their own decisions were described as promoting contentment and enabling independence. Participants expressed a desire to continue making decisions related to their daily and work activities. Seeking help while maintaining strength could also be facilitated by a designated contact person, who played a crucial role in providing support and motivating inner strength, particularly during changes in care or effort. Inner strength, as a force that empowers independence, was also associated with feelings of contentment, satisfaction, and thriving.

Participants shared narratives about maintaining their self-determination in daily life. They strived to avoid being compelled to move out of their home. The way staff worked could either preserve or impede the older adults’ ability to enact independence. One way to enact independence was described as occurring when experienced staff understood how older adults preferred to receive care, without the need for explicit communication. In such cases, it became possible for individuals to feel independent despite receiving a high degree of support from staff.

However, certain situations made it more difficult to enact independence. For example, some staff were experienced as challenging to communicate with, while others employed different working methods. Participants described situations where staff varied in their attitudes towards the older adults. Some staff adhered to their own working methods and showed no intention of adapting to meet the needs expressed by the older adults.

Participants also recounted experiences of being scolded in their own homes and expressed fear of certain staff. Discussing these types of circumstances required a high level of strength and preparedness, and older adults needed to be convinced in their decisions. In some cases, they had to be willing to refrain from receiving care if necessary. Additionally, younger staff were noted for using different working methods, highlighting the need for training to address perceived competence gap.

Instances were exemplified where staff strongly suggested staying indoors on a rainy day without considering the fact that the elderly person may wish to take a walk despite the weather. This lack of understanding was described when participants had to mobilise their strength to express themselves and make uncomfortable decisions. Sometimes the older adults were visited by staff they experienced as demanding in some way—e.g., the “wrong sex” when it came to receiving assistance with intimate care such as showering; summer substitutes who required extensive guidance; or staff who struggled with the Swedish language. To be able to make uncomfortable decisions, the older adults wanted to be convinced about their decision before acting, because of the risk of making staff sad or offended. Mobilising inner strength to enact independence is exemplified in the following quotation where the personnel were unwilling to assist the older adult.

*“Well, there was [a staff] here …who was going to help me with the food. I wanted him to [help me], but that wasn’t his job, he said, his job was just to put it in the microwave. So I told him I didn’t need his help.” (no. 3)*.

### Accepting increasing dependence on others

In summary, participants described getting old and experiencing changes in function and they strived to accept their dependence on others, which in turn meant decreased self-determination. This acceptance of dependency also could sometimes be experienced as a relief.

It was necessary to accept dependence on others because of decreased physical and mental functioning. Some described that it was difficult to accept being dependent on others. Daily life was described as changing over the course of one’s lifetime, and participants described that it could be terribly difficult to grow old. Lack of friends or pets that earlier had been part of life were missed, and they described being dependent on others to fill their need to be social. They also described that it was difficult to engage in activities without accepting help from others. For example, activities like going to the bank, the store, or the library were reduced because of dependence on others and due to the need for expensive transportation. Some described a wish to go out among people and get some sun, but it was difficult to accept that it had to be scheduled. The loss of the opportunities to engage in activities motivated participants to accept help from others.

Some participants described that they lacked the will to decide everything by themselves, instead they accepted dependence on others and felt safe when someone else supported them in the decisions they had made.

*“I’m reliant on others now, but I suppose things are going pretty well… it was hard initially, but this is something I’ve learned from being here five or six years, but I found it hard to be dependent on others initially.” (interview 8)*.

### Experiencing self-determination as shared decision-making

In summary, older adults described that self-determination was influenced by the atmosphere, meaning that the atmosphere could promote or inhibit experiencing self-determination in terms of shared decision-making or in terms of independence. The atmosphere was affected by both physiological milieu, which included interaction and communication, and by the physical environment. Also, staffs’ varied competence affected the atmosphere, and could lead to stressful situations for older adults.

A permissive atmosphere was also described as occurring when staff and older adults were happy with each other and had fun together, which made it easier for participants to ask for the help they needed. Being flexible and satisfied with each other meant that both staff and older adults needed to adapt to the daily situation and the older adult’s health status. According to the physical milieu, having meetings at home; e.g., planning care in a familiar place, was described as promoting self-determination in terms of shared decision-making. The home setting contributed to a sense of security that made it easier to communicate one’s wants and wishes.

An inhibiting atmosphere was described when older adults were not listened to, which meant that they could not decide for themselves, making them feel subservient. Not being listened to was described in connection to situations where older individuals asked for help with small things but were refused by staff because the task was not on the work list. The older adults avoided asking about other things if staff had refused to help them several times. Situations were described where the older adult had an assigned contact person that had never talked to them. That kind of relationship was described as inhibiting the older adult from asking for help and significantly undermined self-determination in terms of shared decision making. Not being listened to was also talked about in relation to language skills. It was described that when people don’t speak Swedish well, you can’t communicate. Not being listened to was also described in relation to overall daily care; e.g., applying lotion to one’s legs. Staff performed the lubrication in their own way, some spread the lotion, while others massaged the lotion, which was not experienced as positive by the older adults.

Older adults’ self-determination was also affected when staff or organisations withheld information about changes in care. It was described that care and activities planned by the staff could cost money that they did not have. Not being listened to was also described in relation to the municipality’s assessment. The older adults described that they sometimes had to live a life that someone else forced them to live. Participants talked about living in an apartment and feeling isolated. They described a wish to eat in a dining room with others. However, in the municipality’s assessment, the criteria for moving to a nursing home with a dining room were not met. It was perceived as bad when others, e.g., the municipality, made decisions about the older adults’ care and life situation that was contrary to their will.

Furthermore, staff competence also affected the psychological milieu, and in turn affected older adults’ ability to experience self-determination as shared decision-making. With some staff, closeness seemed to come naturally and strengthened participants’ self-determination and involvement in care decisions. Other staff were described as lacking competence, which meant that some staff did not care or were not committed to their work, which influenced how decisions were made. Uncommitted people were described as coming in, saying hello, and then saying goodbye. Some stayed for a while, but looked at the clock the entire time, which was perceived as unpleasant and stressful.

One participant describes how she, together with her contact person, is able to participate in the planning of her care, but that the caseworker and her family members do not listen and instead create an atmosphere that can inhibit participation in decision-making. For instance, a situation is described in which the caseworker and family members want the participant to accept help with showering. The quotation below describes one example:

*“It’s good if you get to decide to be involved and make decisions, obviously. I don’t know, I don’t think I need to be involved in making decisions [on everything], everything works so well anyway, that’s all there is to it. I don’t know what else to say.” (interview 10)*.

## Discussion

This study explores older adults’ experiences of self-determination in relation to homecare service. The overall theme *transitioning from self-determination as independence towards self-determination as shared decision-making* permeates all themes and was constructed from the subthemes: *mobilising inner strength to enact independence, accepting increasing dependence on others, self-determination influenced by the atmosphere.*

It is reasonable to believe that thriving and being content protect feelings of independence. Some talked about feeling independent despite a high degree of support from staff. A systematic review [24] provided an overview of older adults’ experiences in decision-making and reported that there are two groups of older adults: those who clearly wish to be involved in the decision-making process, and others who feel content and choose to rely on the expertise of their relatives or health care providers. Informal caregivers were reported to be of great support to older adults in their transitional care decision-making process [24]. Decisions about transitioning from one’s home to an institution has been reported to be the most difficult decision to make for older adults [25].

The results of this study showed that older adults *mobilised inner strengths to enact their independence* and described, for example, a will to decide and manage oneself for as long as possible. The results are similar and confirmed by a qualitative study from Australia that demonstrated that older adults desired to be autonomous in their choices based on their personal preferences, and they had clearly described a fear of losing autonomy [26]. Feelings of reduced autonomy and increased dependency have been shown to have strong relationship to the negative effects of aging as comorbidities, cognitive decline, etc. [24]. One interpretation is that older adults who fight to remain independent both preserve their independence but also prevent comorbidities and cognitive decline. In our study, having a will to decide and manage oneself were described as important, and a motivation was avoiding being forced to move out of their home. A Swedish study [27] concluded that living in a familiar environment is a condition that promotes older adults’ sense of security, and allows habits and routines to be maintained. Self-determination is a vital condition in confirming a person’s self-image. Living at home helped overcome the structural and institutional obstacles to remaining independent and feeling secure.

The theme *accepting increasing dependence on others* emerged from emotionally-charged descriptions and included narratives about involuntarily losing contact with loved ones. Losing loved ones is a natural part of life but still posed a problem, especially for older adults, whose relationships with others will be reduced. Relationships with others has been described as being important in coping in life [26]. In the Australian study, results showed that beneficence strengthened older adults’ sense of competence and relatedness to others. Beneficence was defined as “the sense of doing, or having done, good things fort others” [25, p. 230], and is seen as a basic psychological need. The older adults valued feeling competent and expressed concern about being undervalued in their communities [26]. One interpretation is that being engaged in beneficence could work as protection when losing loved ones to preserve one’s self-determination.

Experiences of being dependent on staff with varied competence could force older adults to act as a teacher to the staff. An integrative review [28] about older adults showed that being involved in their care increased older adults’ satisfaction with their care. However, results showed that older adults had a negative attitude towards their ability to influence their own care in context, such as when scheduling home visits or regarding the continuity of care. Lack of involvement is contradictory to basic psychological needs. Results from a qualitative interview study showed that the care provided by trained nurses with sufficient experience and good communication skills was perceived by participants to be good [29]. Trained nurses were interested in their current health status and adjusted care accordingly for participants, which meant the older adults were treated with respect. The participants in the present study described how a promotive or inhibiting atmosphere could influence shared decision-making. A promotive atmosphere emerged when both parties (older adult and staff) were flexible and satisfied with each other and was described as promoting self-determination. Similar findings were shown in the study from the Czech Republic [29], were findings showed that individual nurses’ attitudes were important to older adults, and such care gave participants reassurance and confidence. The opportunity to participate in care provision was crucial for some of the older adults.

### Methodological consideration

To enhance trustworthiness, all the interviews were conducted by the first author and second author at the older adults’ homes. A strength of the study is that we used the same open-ended interview guide and conducted 15 interviews which provided a variety of experiences of self-determination. A limitation of the study may be that the interviews were performed by two interviewers, which can affect the balance between the informant and interviewers. However, the authors contend that having two interviewers was a strength. This was advantageous because one interviewer could concentrate on leading the interview, while the other could attentively capture various perspectives that might not be perceived by the primary interviewer. Additionally, the interviews were relatively short, and the experience was that several elderly individuals did not have the energy to engage in lengthy conversations. However, they were willing to answer questions afterward if we deemed it necessary to further explore specific areas identified during the analyses process. Regardless of the interview length, the authors assessed that the content was relevant for addressing the formulated purpose. To achieve dependability, all authors discussed every step of the analytical process, and tested the stability of meaning units and categories until consensus was reached. We further, reflected on our findings in relation to the interview text. Krippendorff argues that a text never implies a single meaning [30]. Therefore, our results are only one possible interpretation of the interviews.

## Conclusions

The overall theme *transitioning between self-determination and shared decision-making* permeates all subthemes. The study contributes an increased understanding about older adults’ experiences of self-determination. The results can act as a guide when planning future PCC interventions in HCS contexts and improve HCS’ ability to meet older adults’ needs. To summarise, the older adults’ reflections on their own self-determination was balanced within a context where relationships with other people were highlighted as important for shared decision-making and could preserve older adults’ autonomy and self-esteem.

## Data Availability

The interviews analysed during the current study are not publicly available due to promised confidentiality to the participants. All data analysed during this study are included as results in this article.
